# Adrenergic Inhibition with Dexmedetomidine to Treat Stress Cardiomyopathy during Alcohol Withdrawal: A Case Report and Literature Review

**DOI:** 10.1155/2016/9693653

**Published:** 2016-02-24

**Authors:** Zachary M. Harris, Alvaro Alonso, Thomas P. Kennedy

**Affiliations:** ^1^Tulane University Health Sciences Center, Department of Internal Medicine, New Orleans, LA 70112, USA; ^2^Heart & Vascular Institute, Tulane University School of Medicine, New Orleans, LA 70112, USA; ^3^Tulane University Health Sciences Center, Department of Medicine, Section of Pulmonary Diseases, Critical Care Medicine, and Environmental Medicine, New Orleans, LA 70112, USA

## Abstract

Stress (Takotsubo) cardiomyopathy is a form of reversible left ventricular dysfunction with a heightened risk of ventricular arrhythmia thought to be caused by high circulating catecholamines. We report a case of stress cardiomyopathy that developed during severe alcohol withdrawal successfully treated with dexmedetomidine. The case involves a 53-year-old man with a significant history of alcohol abuse who presented to a teaching hospital with new-onset seizures. His symptoms of acute alcohol withdrawal were initially treated with benzodiazepines, but the patient later developed hypotension, and stress cardiomyopathy was suspected based on ECG and echocardiographic findings. Adjunctive treatment with the alpha-2-adrenergic agonist, dexmedetomidine, was initiated to curtail excessive sympathetic outflow of the withdrawal syndrome, thereby targeting the presumed pathophysiology of the cardiomyopathy. Significant clinical improvement was observed within one day of initiation of dexmedetomidine. These findings are consistent with other reports suggesting that sympathetic dysregulation during alcohol withdrawal produces ideal pathobiology for stress cardiomyopathy and leads to ventricular arrhythmogenicity. Stress cardiomyopathy should be recognized as a complication of alcohol withdrawal that significantly increases cardiac-related mortality. By helping to correct autonomic dysregulation of the withdrawal syndrome, dexmedetomidine may be useful in the treatment of stress-induced cardiomyopathy.

## 1. Background

In the United States, approximately 1.2 million patients are hospitalized annually for manifestations of alcohol abuse [1]. Of these patients, approximately 5%, or 60,000 patients, will develop delirium tremens. In the past, mortality for delirium tremens was as high as 20% [2]. Although current appropriate treatment may reduce mortality to a value as low as 1% [1], recent reports have quoted a mortality of 5–15%, or 3,000–9,000 deaths annually, from this condition [3].

During alcohol withdrawal, atrial and ventricular arrhythmia are common, reflecting the combined influence of direct alcoholic cardiac toxicity and the extreme elevations in circulating catecholamines [1]. In one study, 28% of rats undergoing an alcohol withdrawal protocol experienced spontaneous ventricular arrhythmia and related death [4]. Furthermore, 77% of animals undergoing withdrawal developed ventricular arrhythmia when exposed to exogenous epinephrine, and 31% of these animals died. Despite evidence of significant arrhythmogenicity during withdrawal in animal studies, our understanding of cardiac rhythm disturbance in patients actively withdrawing from alcohol remains limited.

Stress-induced (Takotsubo) cardiomyopathy, also known as transient apical ballooning or “broken heart” syndrome, is an increasingly recognized form of transient left ventricular dysfunction associated with emotional or physical stress. The syndrome was first recognized in a review of five cases and named Takotsubo because the left ventricle at end-systole assumes the shape of the octopus pots of Japanese fishermen in the Hiroshima fish markets, with a round bottom and narrow neck [5]. The syndrome predominantly affects postmenopausal woman (90% of cases) [6] with estimated prevalence of 0.7–2.5% [7]. Emerging evidence supports that adverse myocardial effects of high circulating catecholamines underlie the pathophysiology of stress cardiomyopathy [8]. Because symptoms are similar to those of acute myocardial infarction (e.g., sudden onset of chest pain, moderate increase in troponin levels, and electrocardiographic changes suggestive of ischemia), confirmed absence of coronary artery disease is necessary to establish the diagnosis. In contradistinction to acute plaque rupture, echocardiographic wall motion abnormalities in stress cardiomyopathy typically extend beyond the vascular distribution of a single epicardial artery [9].

Despite an excellent long-term prognosis of afflicted patients, stress cardiomyopathy carries a significant mortality rate in the acute setting (i.e., in-hospital mortality rate of 2%) [10]. Importantly, patients with stress cardiomyopathy have an increased rate of ventricular arrhythmia [11]. We report a case of stress cardiomyopathy occurring during severe alcohol withdrawal that was successfully treated with adjunctive dexmedetomidine and review the literature of stress cardiomyopathy associated with alcohol withdrawal.

## 2. Case Presentation

A 53-year-old African American man was brought in by his wife with new-onset seizures. The patient was unable to provide an accurate history due to altered mental status. Per the patient's wife, the patient had stopped drinking alcohol 24–48 hours prior to admission due to financial constraints, after consuming two pints of whiskey per day for the previous 10 years. The seizures began the evening prior to admission and continued throughout the night. These episodes lasted 1-2 seconds, were tonic-clonic in nature, and were associated with urinary incontinence and foaming at the mouth. His past medical history was significant only for hypertension. He had smoked one pack of cigarettes per day for the past 48 years. Upon arrival, his temperature was 37.1 degrees Celsius, pulse was 94 beats per minute, and blood pressure was 160/98 mmHg. Cardiovascular exam revealed a regular rate and rhythm with normal S1 and S2, abdominal exam disclosed hepatosplenomegaly, and neurologic exam was significant for tremulousness. The patient was disoriented on mental status exam. Mean corpuscular volume was 92.3 fL, platelet count was 58 × 10^3^ per *μ*L, AST was 199 units/L, and ALT was 89 units/L. An ECG done upon arrival revealed a normal sinus rhythm and QTc interval of 546 ms.

Given the recent onset of seizure-like episodes, history of abrupt discontinuation of chronic alcohol use, exam findings, and lab values consistent with ethanol abuse, a diagnosis of acute alcohol withdrawal was suspected. The patient was admitted to the medical intensive care unit. Treatment was initiated with diazepam 5 mg IV every 8 hours and lorazepam 2 mg IV every 1 hour as needed for agitation and anxiety. The patient was also given 100 mg of thiamine, 1 mg of folic acid, and 2 gm of magnesium sulfate IV every 24 hours.

During the 1st hospital day, the patient exhibited witnessed tonic-clonic seizure activity, as well as significant anxiety and agitation. During the 2nd and 3rd hospital day, the patient's mental status fluctuated significantly. Intermittently, the patient was severely agitated, attempting to dislodge his indwelling urinary catheter and requiring 4-point soft restraints. Throughout this period, temperature peaked at 37.4 degrees Celsius, pulse ranged from 71 to 99 beats/minute, and blood pressure ranged from 118 to 154/88–103 mmHg. Diazepam was changed to 7.5 mg PO every 6 hours; however the patient still required lorazepam 2 mg IV every 4 hours for anxiety and agitation.

**Figure 1:**
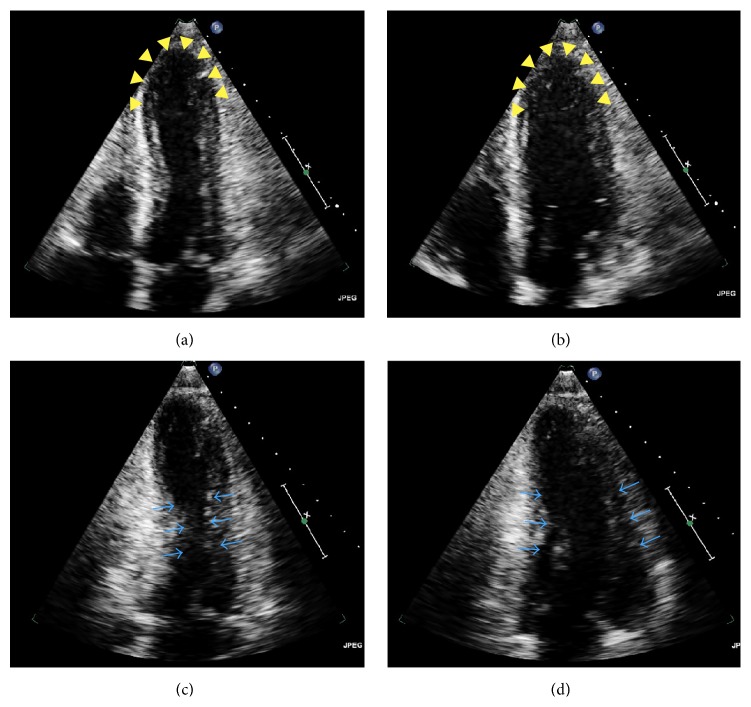
Initial transthoracic echocardiogram in the acute setting revealing features suggestive of stress cardiomyopathy. Systolic (a) and diastolic (b) apical 4-chamber views reveal akinetic apical and mid-anteroseptal segments (arrowheads) with hyperdynamic middle segments. Systolic (c) and diastolic (d) apical 2-chamber views disclose normal wall motion of the mid-cavity (arrows). The circumferential pattern of left ventricular myocardial dysfunction characterized by symmetric wall motion abnormalities involving the septal, anterior, and lateral walls is highly suggestive of stress cardiomyopathy.

**Figure 2:**
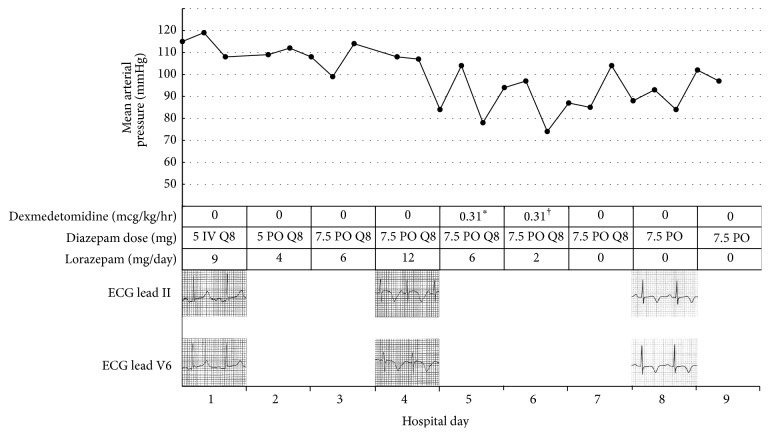
Mean arterial pressure versus time; graph of mean arterial pressure readings against time with corresponding scheduled doses of diazepam, administration of lorazepam, infusion of dexmedetomidine, and ECG changes. ^*∗*^Infusion started at 11:15 AM. Does not include initial loading dose of 1 *μ*g/kg over 10 minutes. ^†^Infusion stopped at 11:11 AM.

On the 4th hospital day, the patient developed hypotension. Blood pressure was 90–100/70–80 mmHg, and pulse was 82–99 beats/minute. The patient was significantly altered and was therefore unable to report subjective symptoms. ECG disclosed symmetric deep coving T waves in leads II, III, aVF, and V2–V6, as well as a prolonged QTc interval of 645 ms. Serum troponin I was elevated at 1.25 ng/mL. Repeat ECGs continued to show symmetric T wave inversions in the inferior limb and lateral chest leads. Serial troponin levels, however, trended downward. An urgent transthoracic echocardiogram revealed multiple left ventricular regional wall motion abnormalities, notably akinesis of the mid-distal anteroseptal, mid-distal anterolateral, mid-distal inferoseptal, mid-distal inferolateral, and apical segments (Figure 1). Of note, right ventricular systolic function was normal, and there was no mitral regurgitation. The left ventricular ejection fraction was estimated to be 40–45%. The clinical instability of the patient precluded emergent coronary angiography. Given the widespread repolarization abnormalities by ECG, diffuse wall motion abnormalities involving myocardial segments circumferentially throughout the entire cardiac apex, presence of an identifiable stressor, and downward trend of cardiac troponins, the diagnosis of stress cardiomyopathy was suspected. The patient continued to require additional doses of lorazepam 2 mg every 2 hours due to agitation and anxiety. On the 5th hospital day, adjunctive treatment with the alpha-2-adrenoreceptor agonist dexmedetomidine was initiated to reduce central sympathetic outflow, directly targeting the presumed pathobiology of the stress cardiomyopathy. A loading dose of 1 *μ*g/kg IV was given over 10 minutes, followed by a maintenance infusion of 0.2 *μ*g/kg/hr titrated by 0.1 *μ*g/kg/hr every 15 minutes to maintain a Richmond Agitation-Sedation Scale of −1. The mean infusion rate was 0.31 *μ*g/kg/hr, administered over a period of 23.93 hours (Figure 2).

**Figure 3:**
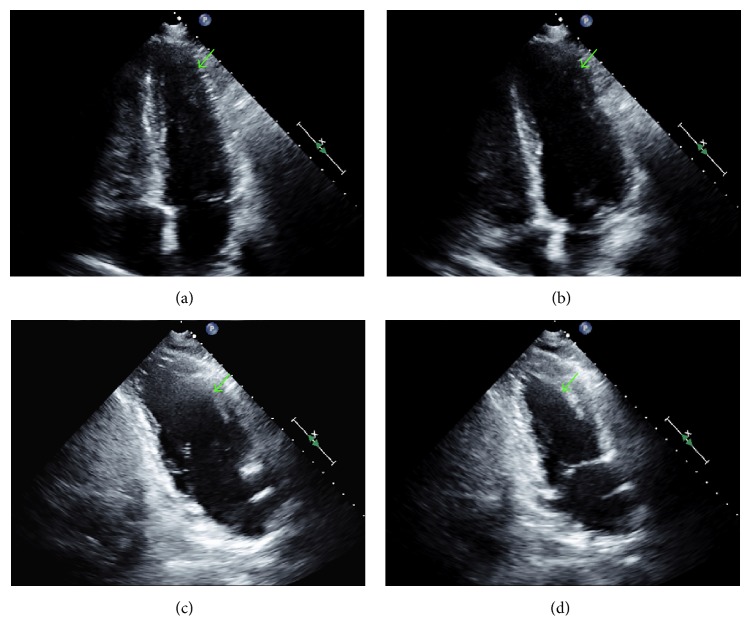
Repeat transthoracic echocardiogram confirming complete recovery of ventricular systolic function and resolution of the acute wall motion abnormalities, supporting the diagnosis of stress cardiomyopathy. Systolic (a) and diastolic (b) apical 4-chamber views and systolic (c) and diastolic (d) apical 2-chamber views reveal normal motion of the ventricular apex (arrows).

On the 6th hospital day, the patient showed marked clinical improvement. Per the patient's family, mental status was at baseline. The patient denied shortness of breath or chest pain. Cardiovascular and pulmonary examinations were unchanged, and neurologic examination revealed no agitation, anxiety, or tremulousness. The patient was awake, alert, and oriented to person, place, and time. Dexmedetomidine therapy was weaned by 0.1 *μ*g/kg/hr every 15 minutes. The patient was treated thereafter with tapering doses of diazepam PO, and no additional doses of lorazepam were required. A coronary angiogram was offered to the patient to rule out coronary artery obstruction; however the patient refused the procedure. One month after hospitalization, repeat ECG revealed improvement of the T wave inversions when compared to the ECGs during the event. The QTc was shortened to 495 ms. Coronary CT angiography was performed, which revealed absence of coronary artery disease. Repeat ECG two months later revealed a normal sinus rhythm with no significant T wave inversions. Subsequent echocardiography performed 14 months after the event revealed resolution of the acute regional wall abnormalities and confirmed a full recovery of ventricular systolic function, further supporting the diagnosis of stress cardiomyopathy (Figure 3).

## 3. Discussion

This case report describes a patient who developed stress cardiomyopathy during severe alcohol withdrawal. For this patient, we reasoned that high sympathetic outflow from the withdrawal syndrome caused stress cardiomyopathy. Although we were unable to angiographically confirm absence of acute plaque rupture at the time of the event due to clinical instability, we believe that the diffuse circumferential pattern of ventricular wall motion abnormalities extending beyond a single epicardial vascular territory as revealed by transthoracic echocardiography and global ECG abnormalities not confined to a particular anatomic distribution strongly argue against myocardial ischemia as the cause of the patient's presentation. Moreover, the coronary CT angiogram excluding the presence of coronary artery disease and the resolution of ECG changes further support the diagnosis and fulfill the proposed Mayo Clinic criteria for stress-induced cardiomyopathy [21]. Although repeat echocardiography was not obtained until 14 months after the event, demonstration of full recovery of ventricular function and complete resolution of the acute wall motion abnormalities strongly support the diagnosis according to the recent diagnostic criteria proposed by the Heart Failure Association of the European Society of Cardiology [22]. During the event, we targeted central alpha-2-adrenergic receptors with dexmedetomidine to curtail excessive catecholamine release, thereby disrupting the presumed pathophysiologic cause of the cardiomyopathy.

Increasing evidence suggests that catecholamine-mediated effects are responsible for the development of stress cardiomyopathy [8]. Exposure to endogenous (emotional, related to preexisting condition) or exogenous (trauma, surgical procedure, and exacerbation of preexisting condition) stress and increased sympathetic activity have been reported in most cases of stress cardiomyopathy. Reports have confirmed increased catecholamine levels in the serum of patients who developed stress cardiomyopathy [23, 24]. Stress cardiomyopathy is characterized by pathohistological changes similar to catecholamine cardiotoxic effects observed in animals and humans, including increased production of extracellular matrix proteins, contraction band necrosis (a unique form of myocyte injury highly specific for prolonged exposure to elevated levels of catecholamines), and mild neutrophil infiltration [25–27]. Furthermore, there is evidence that supraphysiologic catecholamine levels act through cardiomyocyte beta-adrenoreceptors to produce calcium-regulatory protein gene expression disturbances [28–31]. Calcium dysregulation has been demonstrated in stress cardiomyopathy, and increased densities of calcium-regulatory proteins, such as sarcolipin, have been found in cardiomyocytes of patients with stress cardiomyopathy [32]. The effects of catecholamine-induced calcium disturbances in the pathogenesis of stress cardiomyopathy are currently being explored.

Severe alcohol withdrawal is a hyperadrenergic state associated with elevated levels of circulating catecholamines and as such produces ideal pathobiology for the development of stress cardiomyopathy. The pathophysiology of acute alcohol withdrawal involves the compensatory neurobiological changes that occur in the setting of chronic alcohol ingestion [33]. Alcohol produces a generalized depressant effect on the central nervous system mainly through activation of the gamma aminobutyric acid (GABA) pathway. Acute ingestion of the drug boosts GABA activity, resulting in the anticonvulsant, sleep-inducing, antianxiety, and muscle relaxation effects of all GABA-boosting agents [34]. With chronic alcohol exposure, there is a compensatory upregulation of both GABA_A_ receptors and excitatory glutamate N-methyl-D-aspartate (NMDA) receptor subunits. When alcohol is acutely withdrawn in the setting of long-term constant exposure (desistance), high NMDA and low GABA activity produce a state of central nervous system arousal. This molecular milieu is responsible for the initial clinical manifestations of acute alcohol withdrawal, including tremors, insomnia, vivid dreams, anorexia, anxiety, nausea and vomiting, paroxysmal sweating, weakness, and myalgia [1]. Notably, acute withdrawal produces unopposed catecholaminergic activation and decreased central inhibition that result in autonomic instability. Increased levels of urine, plasma, and cerebrospinal fluid norepinephrine and its metabolite, 3-methoxy-4-hydroxyphenylglycol (MHPG), are seen in alcoholics undergoing withdrawal [35–38]. Tachycardia, systolic hypertension, and a coarse tremor of the extended hands and tongue are common. Two–5% of patients in acute alcohol withdrawal will develop delirium tremens, a form of severe, late withdrawal lasting 5–7 days, which carries a high mortality rate [39]. During severe withdrawal and delirium tremens, elevated levels of circulating catecholamines are associated with increased cardiac output, stroke volume, and oxygen consumption [38, 40]. High circulating catecholamines and the resultant adverse effects on cardiomyocytes help explain the development of stress cardiomyopathy in patients undergoing severe withdrawal.

Although atrial and ventricular arrhythmia occur frequently during acute alcohol withdrawal, the pathophysiology of arrhythmogenesis during the withdrawal syndrome remains poorly understood [1, 4]. Stress cardiomyopathy may help explain increased arrhythmogenicity observed in alcohol withdrawal. Reports have demonstrated a significant (9%) incidence of ventricular arrhythmia during stress cardiomyopathy [11]. To the best of our knowledge, there have been at least nine published reports of stress cardiomyopathy occurring during acute alcohol withdrawal (Table 1) [12–20]. In our review of these cases, six out of the nine patients exhibited QT prolongation via ECG, and two of the patients developed ventricular arrhythmia (both degenerated to ventricular fibrillation). Two of the nine patients developed hypotension, which required vasopressor support. Confounding variables in these cases included recent usage of cocaine in one of the patients who developed ventricular fibrillation and major surgery requiring reintubation. Long-standing alcohol abuse by itself produces cardiotoxic effects and is a risk factor for sudden cardiac death [41]. Nonetheless, by increasing the likelihood of arrhythmia, stress cardiomyopathy may significantly contribute to mortality in acute alcohol withdrawal, especially in patients with a predisposition to cardiac arrhythmia from chronic alcohol abuse.

Emerging evidence supports that certain demographic and clinical features have significant prognostic implications on the clinical course of patients hospitalized for stress cardiomyopathy. According to one study, advanced age (≥75 years) appears to be an independent risk factor for major adverse events, including all-cause death, acute heart failure, life-threatening arrhythmia, stroke, and cardiogenic shock [42]. Patients of advanced age hospitalized for stress cardiomyopathy presented with significantly worse glomerular filtration rates, were more apt to receive inotropic agents during hospitalization, and did not recover cardiac systolic function at the time of discharge as efficiently when compared with younger patients.

Given its widespread availability and feasibility in the acute setting, echocardiography is frequently the first noninvasive imaging modality used to assess patients with stress cardiomyopathy [43]. In this case, transthoracic echocardiography played a central role in establishing the diagnosis of stress cardiomyopathy and furthermore provided important prognostic information in the acute setting. In contrast to acute myocardial infarction caused by occlusion of an epicardial artery, where wall motion abnormalities are typically limited to regions supplied by a single occluded vessel, the echocardiographic topography of left ventricular dysfunction in stress cardiomyopathy is characterized by a diffuse, circumferential wall motion abnormality pattern typically involving myocardial segments spanning multiple vascular territories [43, 44]. Furthermore, there is evidence that regional wall motion abnormalities in stress cardiomyopathy tend to involve territories partially supplied by the right coronary artery to a significantly greater extent compared with anterior ST-elevation myocardial infarction [44]. Echocardiographic parameters used to quantify the anatomic extent of regional wall motion abnormalities, such as the left ventricular wall motion score index and the number of territories with regional wall motion abnormalities, are useful correlates with high sensitivities and specificities in the prediction of stress cardiomyopathy [44]. Immediate and accurate echocardiographic assessment of regional wall motion abnormalities is therefore crucially important in the differential diagnostic algorithm of patients presenting with clinical features suggestive of stress cardiomyopathy. In the presented case, transthoracic echocardiography revealed a distinctive pattern of contractility characterized by symmetrical regional wall motion abnormalities extending into the territory of multiple epicardial arteries and hypercontractility of the basal segments of the heart. This pattern was sufficiently characteristic to significantly raise the diagnostic pretest probability of stress cardiomyopathy, especially given the surrounding demographic and clinical context.

Moreover, a number of echocardiographic correlates provide prognostic information important in the clinical management of patients with stress cardiomyopathy [45]. In a recent multicenter prospective study, echocardiographic correlates including ventricular ejection fraction, wall motion score index, *E*/*e*′ ratio, left ventricular outflow obstruction, pulmonary artery systolic pressure, right ventricular involvement, and reversible moderate-to-severe mitral regurgitation were associated with adverse hospitalization events (e.g., acute heart failure, cardiogenic shock, and in-hospital mortality) in patients diagnosed with stress cardiomyopathy [45]. At multivariate analysis, left ventricular ejection fraction (HR: 0.92; 95% CI: 0.89–0.95; *p* < 0.001), *E*/*e*′ ratio (HR 1.13; 95% CI: 1.02–1.24; *p* = 0.011), and reversible moderate-to-severe mitral regurgitation (HR: 3.25; 95% CI 1.16–9.10; *p* = 0.025) were independent correlates of major adverse events. These features on initial echocardiogram in the acute setting should therefore raise clinician alertness to patients at risk of the development of major in-hospital adverse events, ultimately affecting clinical management of this high-risk patient subset. Right ventricular involvement is an echocardiographic correlate of stress cardiomyopathy syndrome severity that deserves special emphasis. In a study of 339 primarily female patients with stress cardiomyopathy, the presence of right ventricular involvement within 24 hours of symptom onset was an independent predictor of adverse events (HR: 2.327; 95% CI: 1.151–4.706; *p* = 0.019), albeit the prevalence of extensive right ventricular involvement was relatively low (<17%) [46]. In the presented case, the absence of specific features on the initial echocardiogram (e.g., left ventricular outflow obstruction, reversible moderate-to-severe mitral regurgitation, and right ventricular involvement) portended a favorable clinical prognosis.

Benzodiazepine therapy targeting GABA_A_ receptors is the mainstay of treatment for acute alcohol withdrawal. This treatment strategy targets the imbalance between GABA neuroinhibitory activity and NMDA neuroexcitatory activity observed in alcohol withdrawal. In severe cases unmitigated by GABA-targeted agents, therapy directed toward dysautonomia may be beneficial [47]. Normally, central alpha-2-adrenoreceptors inhibit the firing of presynaptic norepinephrine neurons. In chronic alcohol users and in actively withdrawing patients, alpha-2-adrenergic receptor signaling may become less sensitive, resulting in dysregulation of norepinephrine and epinephrine release. This mechanism was identified in studies showing decreased blood pressure response to alpha-2-adrenoreceptor agonist therapy in patients actively withdrawing from alcohol [47]. Alpha-2-adrenergic agonists, such as clonidine, have been used to treat alcohol withdrawal, although their use has fallen off with the rise of the benzodiazepines. The alpha-2-adrenergic agonist dexmedetomidine, a novel agent with receptor specificity 8 times that of clonidine, has stimulated interest for its potential use in the treatment of acute alcohol withdrawal [48]. Dexmedetomidine aids in the treatment of active withdrawal by restoring alpha-2-adrenergic modulation, thus helping to correct catecholamine overproduction. Because it does not act on GABA_A_ or opioid receptors, dexmedetomidine has the added benefit of conferring sedation without respiratory compromise [49], an effect that helped lead to the agent's approval for sedation by the Federal Drug Administration. Randomized clinical trials studying the utility of dexmedetomidine in the treatment of patients undergoing severe withdrawal are currently in effect. As in the presented case, the treatment of stress cardiomyopathy and prevention of subsequent cardiac complications may represent a beneficial, potentially unrecognized role of central alpha-2-adrenergic agonists like dexmedetomidine in acute alcohol withdrawal.

## 4. Conclusions

Despite our knowledge of cardiovascular dysregulation with abrupt cessation of alcohol, the pathophysiology of arrhythmogenesis in alcohol withdrawal remains poorly understood. In the case presented, the patient developed stress cardiomyopathy during acute alcohol withdrawal and was successfully treated with adjunctive dexmedetomidine. Our literature review supports the concept that supraphysiologic levels of catecholamines lead to the development of stress cardiomyopathy in alcohol withdrawal and that modulation of disorganized catecholamine release with alpha-2-adrenergic agonists may have an important role in treatment. Our review of nine published cases indicates that stress cardiomyopathy may be an important determinant of malignant arrhythmia in patients actively withdrawing from alcohol. Furthermore, this case demonstrates the central role of transthoracic echocardiography in the diagnostic workup and prognosis of patients suspected to have stress cardiomyopathy. Clinicians should be aware of stress cardiomyopathy as a complication of alcohol withdrawal that may significantly increase cardiac-related mortality. Studies examining the cardioprotective role of alpha-2-adrenoreceptor agonists in the treatment of alcohol withdrawal are warranted.

## Figures and Tables

**Table 1:** Case reports of stress cardiomyopathy in patients with acute alcohol withdrawal. VTach, ventricular tachycardia; VFib, ventricular fibrillation; CXR, chest X-ray; CIWA, Clinical Institute Withdrawal Assessment of Alcohol scale.

Age/sex	Onset (from last drink)	Clinical features of withdrawal	Presenting features of stress cardiomyopathy	Notable case features	Reference
64/M	At least 5 days	Not described in case report	Decline in level of consciousness, sustained VTach with degeneration to VFib, cardiopulmonary arrest; subsequent ST segment elevation and later T wave inversion	QT prolongation on admission; patient required cardiopulmonary resuscitation	[12]

49/F	Not described in case report	Withdrawal seizure after episode of acute intoxication (time between events not described in case report)	Decreased level of consciousness, decreased O_2_ saturation, rapid hypotension, infiltrate on CXR, ST elevation, and T wave inversion	—	[13]

25/F	Not described in case report	Seizure episode	Torsades de pointes with degeneration to VFib (in route to hospital), T wave inversion (3 hours after resuscitation) with QT prolongation	Cocaine use 3 days prior to seizure	[14]

61/M	36 hours	Not described in case report	Chest pain radiating to jaw, tachycardia, ST elevation, and T wave inversion	—	[15]

63/M	6-7 days	Grand mal seizure 3 days after alcohol cessation	Severe dyspnea, pulmonary edema, T wave inversion, QT prolongation	Resolution of stress cardiomyopathy confirmed at 10 weeks	[16]

56/M	5 days after hospitalization	Confusion, severe asthenia, anorexia, tremor	Tachycardia, decreased O_2_ saturation, pulmonary edema, orthopnea, pathologic Q waves, elevated troponin I (1.08 ng/mL)	3 days after onset of stress cardiomyopathy, ECG showed diffuse T wave inversion and QT prolongation	[17]

57/F	<24 hours	2 episodes of seizures and confusion the morning after a night of binge drinking, fever, tachycardia, agitation, diaphoresis, tremulousness,	Hypotension, T wave inversion, QT prolongation, elevated troponin I (4.075 micrograms/L), subsequent elevated jugular venous pressure and peripheral edema	History of alcohol-related seizures; patient required vasopressor support; reversal of left ventricular wall motion abnormalities 12 days prior to admission	[18]

45/F	96–120 hours	Epigastric pain, nausea and vomiting 72 hours after discontinuation of alcohol, tremulousness, tachycardia, CIWA of 9	T wave inversion, troponin elevation (0.974 ng/mL)	—	[19]

57/F	>10 days	Intense agitation, tachycardia, tachypnea	Tachycardia, tachypnea, pulmonary edema, Q waves, ST elevation, T wave inversion, subsequent ECG revealed diffuse T wave inversion and QT prolongation	Patient admitted for elective thoracotomy; patient was reintubated after procedure due to hypoxemic respiratory failure; dexmedetomidine used in the treatment of withdrawal symptoms; patient later developed cardiogenic shock and required vasopressors and intra-aortic balloon pump	[20]
